# Targeting the osteoimmune microenvironment to prevent regulated chondrocyte death in osteoarthritis: therapeutic potential of natural products

**DOI:** 10.3389/fcell.2026.1797496

**Published:** 2026-06-26

**Authors:** Ruofan Liu, Xinyang Wang, Ziheng Zhu, Yunfei Li, Chuanbing Huang

**Affiliations:** 1 The First Affiliated Hospital of Anhui University of Traditional Chinese Medicine, Hefei, China; 2 School of Chinese Medicine, Bozhou University, Bozhou, China; 3 Key Laboratory of Xin’an Medicine, Ministry of Education, Anhui University of Chinese Medicine, Hefei, China

**Keywords:** ferroptosis, metabolic reprogramming, nanomedicine, natural products, osteoarthritis, osteoimmune microenvironment, regulated cell death

## Abstract

Osteoarthritis (OA) is increasingly recognized as a whole-joint disease driven by a dysregulated osteoimmune microenvironment, rather than mere mechanical wear. Despite the high prevalence of OA, current pharmacotherapies are largely palliative, failing to halt cartilage degeneration or reverse chondrocyte death. Consequently, the development of Disease-Modifying Osteoarthritis Drugs (DMOADs) targeting the underlying pathology remains an urgent unmet need. Natural products, distinguished by their pleiotropic nature, have emerged as promising candidates capable of functioning as “network regulators” to restore joint homeostasis. This review systematically elucidates the multi-dimensional mechanisms by which natural products prevent regulated cell death (RCD) in chondrocytes. We highlight their capacity to orchestrate upstream immune responses by repolarizing synovial macrophages, blocking inflammatory cascades (e.g., NLRP3 inflammasome), and restoring downstream cellular defenses. Special attention is given to emerging mechanisms, including the inhibition of ferroptosis and the reversal of metabolic reprogramming via the modulation of the HIF-1α/HIF-2α switch and the NAD+/SIRT1 axis. Furthermore, we extend the discussion to the subchondral bone, emphasizing the restoration of the osteochondral unit. To bridge the gap between preclinical success and clinical translation, we discuss current challenges such as bioavailability and propose future strategies involving biomimetic nanodelivery systems and precision medicine based on OA endotypes. Collectively, this review provides a comprehensive framework for utilizing natural products to rewire the osteoimmune microenvironment, highlighting their potential as promising candidates for future DMOAD development.

## 1 Introduction

Osteoarthritis (OA) is a prevalent global disease affecting approximately 600 million people worldwide, imposing a significant economic burden across various regions (Cao et al., 2024; Collaborators, 2023). Its incidence is closely correlated with aging (Prieto-Alhambra et al., 2014; O'Brien and McDougall, 2019), making OA a leading cause of disability and reduced quality of life among the elderly (Abramoff and Caldera, 2020; Chen et al., 2026). Historically, OA was characterized primarily as a “wear and tear” disease, a hypothesis supported by the strong correlation between disease incidence and age (Prakash et al., 2024). However, with advancing knowledge of inflammatory pathways and rheumatic diseases, OA is now understood as a whole-joint disease driven by low-grade chronic inflammation, involving complex cytokine networks similar to those seen in other rheumatic conditions (Wojdasiewicz et al., 2014; Greene and Loeser, 2015).

While the high prevalence and severity in weight-bearing joints like the knee and hip initially supported the biomechanical wear theory (Long et al., 2022), this perspective fails to explain systemic susceptibility to OA (Marshall et al., 2018; Favero et al., 2022). Consequently, current research has shifted focus towards complex pathogenic mechanisms involving synovial inflammation and metabolic dysregulation (Dieppe and Lohmander, 2005). Although cartilage, synovium, meniscus, and ligaments are all affected during OA progression, current evidence suggests that synovium-joint crosstalk is a critical pathological component, playing a central regulatory role in inflammation amplification, pain generation, and structural progression (Kong et al., 2025). Damage-associated molecular patterns (DAMPs) derived from cartilage matrix degradation (e.g., collagen fragments, aggregates) are key triggers. Specifically, cartilage debris can trigger a “foreign body-like” reaction in the synovium (Lambert et al., 2020), activating macrophages to release inflammatory cytokines. Synovitis represents the inflammatory manifestation of the dysregulated osteoimmune microenvironment on the articular side; it deeply participates in subchondral bone remodeling and OA progression by regulating key signals such as macrophage polarization and RANKL (Zhang K. et al., 2025). This inflammation cascade driven by the synovium may contribute to the continuous deterioration of the intra-articular osteoimmune microenvironment (Hu W. et al., 2021). The worsened microenvironment, through persistent cytokine stimulation, aberrant immune signaling activation, and uncoupled bone-cartilage metabolism, collectively drives chondrocytes from homeostasis maintenance toward programmed death. This represents a central pathological driver underlying the irreversible progression of cartilage degeneration in OA.

Given the generally slow progression of OA, current treatments predominantly rely on oral non-steroidal anti-inflammatory drugs (NSAIDs), corticosteroids, and injectable biological agents. NSAIDs and corticosteroids target only downstream symptoms like pain and inflammation, while biologics primarily aim to block single inflammatory targets, which often proves insufficient in the context of the low-concentration, multi-factor inflammation characteristic of OA (Richard et al., 2023). Currently, no true Disease-Modifying Osteoarthritis Drug (DMOAD) has been identified (van Galen et al., 2025). Natural products, by regulating synovial inflammation, macrophage polarization, and osteoimmune signaling networks via multiple targets, can remodel the osteoimmune microenvironment. This indirectly but sustainably inhibits chondrocyte death, offering a crucial strategy for the disease-modifying treatment of OA and providing new possibilities in the exploration of DMOADs.

In this review, osteoimmunology is defined as the study of the bidirectional interactions between the skeletal and immune systems, with a particular focus on immune cell populations (e.g., macrophages, T cells), their spatial distribution within joint tissues, and signaling pathways mediating bone-cartilage-immune crosstalk (e.g., NF-κB, cytokine networks). In this work, we adopt a relatively narrow definition, emphasizing immune cell-driven regulation of the joint microenvironment, rather than general inflammatory or metabolic processes not specifically mediated by immune components.

Importantly, emerging evidence suggests that osteoimmune dysregulation is not only a driver of inflammation but also a key upstream regulator of various forms of chondrocyte-regulated cell death, including apoptosis, pyroptosis, ferroptosis, and autophagy-dependent cell death. Immune cell-derived cytokines, metabolic mediators, and oxidative signaling collectively determine chondrocyte fate, thus directly linking osteoimmune processes to cartilage degeneration.

Crucially, the osteoimmune microenvironment acts as a crucial regulator of chondrocyte fate. Specific immune components, such as M1 macrophages and Th17 cells, initiate distinct cell death programs: pro-inflammatory cytokines drive pyroptosis via the NLRP3 axis, while immune-driven oxidative bursts trigger mitochondrial apoptosis and ferroptosis. This review provides a focused mapping of how natural products intercept these specific immune-to-RCD cascades.

## 2 The osteoimmune microenvironment in OA: a trigger for regulated cell death

Notably, chondrocyte death in OA is not a uniform process but involves multiple modes of Regulated Cell Death (RCD), including Apoptosis, Pyroptosis, and the recently identified Ferroptosis. These death modalities intertwine within the osteoimmune microenvironment, collectively driving cartilage loss.

### 2.1 Synovial macrophages and the inflammatory milieu: drivers of microenvironmental imbalance

Unlike the intense autoimmune response typical of Rheumatoid Arthritis (RA), the immune microenvironment of OA is characterized by unique “long-term, low-grade chronic inflammation” (Knights et al., 2023). Although the absolute concentration of inflammatory mediators is lower, this persistent inflammatory stress is sufficient to disrupt intra-articular immune homeostasis. This microenvironmental alteration is not a transient fluctuation but a pathological ecological remodeling that gradually undermines chondrocyte viability, sensitizing them to death signals (Zhang et al., 2020). The core driver lies in the polarization imbalance of synovial macrophages. Upon stimulation by DAMPs generated from cartilage wear, synovial resident macrophages tend to polarize towards the pro-inflammatory M1 phenotype. These M1 macrophages continuously secrete IL-1α, together with other key pro-inflammatory cytokines such as IL-1β and TNF-α, forming a positive feedback loop with osteoclasts in the subchondral bone, which further accelerates the degeneration of the osteochondral unit (Hu and Wang, 2025; Li M. et al., 2022). Given this, therapies targeting single molecules are often ineffective. Targeting the remodeling of the microenvironment—specifically by inhibiting M1 polarization, clearing inflammatory mediators, and restoring immune-cartilage homeostasis—represents a more promising comprehensive therapeutic strategy.

### 2.2 Adaptive immune dysregulation: the Th17/Treg imbalance

While macrophages orchestrate innate immunity, recent single-cell RNA sequencing (scRNA-seq) studies indicate that adaptive immune cells, particularly T lymphocytes, represent a prominent immune subset in the OA synovium (Zhang X. et al., 2025). OA progression is closely linked to a breakdown in peripheral tolerance, characterized by an imbalance between pro-inflammatory T helper 17 (Th17) cells and immunosuppressive Regulatory T (Treg) cells (Pemmari et al., 2021; Zhu and Paul, 2008).

Under homeostatic conditions, Treg cells maintain immune tolerance by suppressing excessive inflammatory responses via the secretion of IL-10 and TGF-β (Zong et al., 2024). However, in the OA microenvironment, this balance is disrupted. The distinct cytokine milieu (rich in IL-6 and IL-1α) drives the differentiation of naive CD4^+^ T cells towards the pathogenic Th17 lineage while suppressing Treg differentiation or inducing Treg instability. Th17 cells secrete IL-17A, a potent catabolic cytokine that acts directly on chondrocytes to suppress Collagen II synthesis and upregulate MMP-13 expression. Furthermore, IL-17A synergizes with TNF-α to amplify the inflammatory cascade in synoviocytes (Guo et al., 2025; Li et al., 2017). Conversely, although Treg cells are present in OA joints, they often exhibit an “exhausted” phenotype or functional plasticity, converting into Th17-like Treg cells (Foxp3+IL-17+) that lose their suppressive capacity and contribute to pathology (Akhter et al., 2023). This shift from a “regulatory” to a “destructive” adaptive immune profile constitutes a critical, yet often overlooked, trigger for chondrocyte apoptosis (Wen et al., 2024).

### 2.3 Cytokine imbalance and the inflammatory cascade

“Cytokine storm” traditionally refers to an uncontrolled, cascading release of massive amounts of inflammatory factors over a short period, leading to systemic inflammation and organ failure (Fajgenbaum and June 2020). Although the chronic cytokine imbalance in OA differs from this acute, uncontrolled “cytokine storm” (Karki et al., 2026), the pathological microenvironment it establishes progressively erodes cartilage (Motta et al., 2023).

As mentioned above, the persistent osteoimmune imbalance, specifically M1 macrophage dominance, serves as the direct trigger for pyroptosis. M1 macrophages continuously release inflammatory factors; synovium-derived IL-1α and TNF-α selectively bind to chondrocyte surface receptors (IL-1R and TNFR), activating the NF-κB pathway. This not only initiates pro-inflammatory gene transcription but also stimulates the cell to produce large amounts of Reactive Oxygen Species (ROS) and Nitric Oxide (NO). Excessive ROS further activates the NLRP3 inflammasome and Caspase-1, leading to pyroptosis and inducing an autocrine loop of inflammatory cytokines, thereby forming a self-amplifying inflammatory network within the joint microenvironment (De Roover et al., 2023).

### 2.4 Hypoxia and HIF imbalance: the metabolic switch

In addition to the inflammatory chronic cytokine imbalance, the rapid proliferation of immune cells and the avascular nature of cartilage create a distinct hypoxic microenvironment (Zheng et al., 2021; Jiang et al., 2025). This hypoxic stress drives a transcriptional shift. Hypoxia-Inducible Factor-1 (HIF-1α) is a core transcription factor maintaining chondrocyte homeostasis (Pfander et al., 2006). HIF-1α not only promotes the synthesis of Collagen II and proteoglycans but also directly activates mitophagy via BNIP3 to clear damaged mitochondria and inhibit ROS production. However, in the OA microenvironment, this balance is disrupted by inflammation, driving a pathological transition from HIF-1α to HIF-2α. Unlike HIF-1α, which maintains homeostasis (Zhang J. et al., 2025), NF-κB-induced HIF-2α is a potent inducer of catabolism (Li W. et al., 2022). Overexpressed HIF-2α directly binds to the promoters of MMP-13 and ADAMTS-5 to accelerate matrix degradation and enhances cellular sensitivity to apoptosis via the Fas/FasL pathway (Zhang et al., 2015). This “metabolic reprogramming” at the transcriptional level, together with the aforementioned inflammation and oxidative stress, constitutes the core targets for natural products to exert their “network regulation” effects (Zhang et al., 2024).

Thus, OA is not merely the result of immune disorder and oxidative stress; the transcriptional reprogramming biased from HIF-1α to HIF-2α constitutes a critical link in the deterioration of the osteoimmune microenvironment. OA can be viewed as a disease of maladaptive transcriptional reprogramming driven by chronic inflammation, hypoxia, and oxidative stress.

It is important to note that the roles of HIF-1α and HIF-2α are highly complex; their protective or pathogenic effects can vary significantly depending on the specific cell type, disease stage, and experimental model utilized.

Given the network nature of transcriptional reprogramming, the multi-target effects of natural products offer unique advantages. Their role in interfering with transcriptional reprogramming is gaining attention, and their combined intervention in oxidative stress and inflammatory pathways holds significant potential as an ideal strategy for restoring the cartilage microenvironment.

### 2.5 Oxidative stress: the mitochondrial apoptosis pathway

Metabolic reprogramming driven by HIF-2α and cytokine signaling ultimately converges on the mitochondria. The distinct hypoxic and inflammatory environment triggers a massive accumulation of ROS. Following the inflammatory cascade, NF-κB-induced excessive ROS constitutes a second hit to chondrocytes (Lepetsos et al., 2019). Mitochondria are not only the cellular powerhouses but also the primary targets of ROS attacks (Ryu et al., 2012). Persistent oxidative stress directly modifies mitochondrial membrane lipids and proteins, leading to the collapse of the mitochondrial transmembrane potential and the blockade of ATP synthesis, plunging the cell into an energy crisis while initiating the apoptotic program. This process is tightly regulated by Bcl-2 family proteins. Under ROS stimulation, anti-apoptotic proteins are downregulated, while pro-apoptotic proteins undergo conformational changes and translocate to the outer mitochondrial membrane.

This imbalance leads to Mitochondrial Outer Membrane Permeabilization (MOMP). MOMP is the irreversible point of no return, leading to the leakage of the pro-apoptotic factor, Cytochrome C, into the cytoplasm. Cytochrome C binds with Apaf-1 and Pro-caspase-9 to assemble the apoptosome, subsequently activating Caspase-9 and downstream effectors Caspase-3/7. As the terminal executors of the apoptotic cascade, Caspase-3/7 specifically cleave key intracellular substrates such as cytoskeletal proteins and DNA repair enzymes, ultimately resulting in cytoskeletal disintegration, chromatin condensation, and irreversible programmed cell death (Iantomasi et al., 2025; Hwang and Kim, 2015).

### 2.6 Autophagy suppression: collapse of defense mechanisms

Autophagy is an intrinsic survival mechanism in chondrocytes that maintains cellular homeostasis by degrading damaged organelles and misfolded proteins via the lysosomal pathway (Pierrefite-Carle et al., 2015; Ohsumi, 2014; Mizushima and Komatsu, 2011). While autophagy serves as a primary defense mechanism, it often fails during OA progression (Wang et al., 2023). In the inflammatory phase of OA, chondrocytes compensatorily enhance autophagy flux to counter inflammation and ROS-induced damage. However, with the accumulation of inflammatory cytokines and the overactivation of signaling pathways, particularly the PI3K/Akt/mTOR pathway, the activity of the autophagy initiation complex ULK1 is potently inhibited. Under the cumulative effect of multiple factors, lysosomal function is impaired, leading to a blockade of autophagy flux.

**FIGURE 1:**
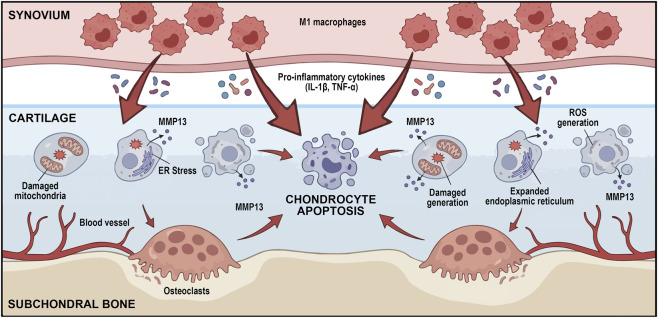
Pathological mechanisms of the osteoimmune microenvironment in OA. Proposed pathway hierarchy in the pathological progression: Upstream immune triggers → inflammatory and metabolic stress→ RCD pathway.

Once this autophagic defense is breached, damaged mitochondria accumulate, exacerbating ROS production and metabolic waste. Overwhelmed by this structural and oxidative stress, chondrocytes initiate the Caspase cascade, ultimately culminating in irreversible regulated cell death (Andrei et al., 2024; Valenti et al., 2021) (Figure 1).

Overall, these processes should not be viewed as isolated pathological events. Instead, macrophage polarization, adaptive immune imbalance, oxidative stress, HIF-dependent metabolic reprogramming, and autophagy dysfunction collectively form an interconnected osteoimmune network that ultimately leads to regulated chondrocyte apoptosis.

## 3 Molecular mechanisms of natural products in regulating the microenvironment

In this review, oxidative stress, HIF imbalance, mitochondrial dysfunction and autophagy are discussed only when they are secondary events initiated or amplified by osteoimmune dysregulation.

Given the limitations of current clinical therapies in reversing OA pathology, there is a critical need for DMOADs that target the core mechanisms of cartilage degradation (Kloppenburg et al., 2025). Natural products, distinguished by their unique multi-target and multi-level pharmacological properties, are emerging as breakthroughs in this field (Xu et al., 2025). However, most existing reviews focus on single compounds or general anti-inflammatory effects. Few studies have systematically elucidated how these natural compounds regulate the osteoimmune microenvironment and reverse metabolic reprogramming via multiple pathways to prevent chondrocyte apoptosis. This section systematically elucidates how natural products systemically regulate the osteoimmune microenvironment. We will explore their capacity to function as network regulators by orchestrating immunomodulation, blocking inflammatory cascades, restoring redox balance, reprogramming metabolism, and restoring autophagy. By dissecting these mechanisms, we aim to provide solid mechanistic evidence for how these compounds restore microenvironmental homeostasis and block chondrocyte regulated cell death.

### 3.1 Modulation of macrophage polarization

Macrophages play a central role in shaping the osteoimmune microenvironment of OA. Pro-inflammatory M1 macrophages increase cytokine production, while M2 macrophages promote tissue repair and inflammation resolution. Several natural products have been shown to effectively regulate macrophage polarization in skeletal and synovial diseases, providing an upstream intervention to inhibit downstream inflammatory and degenerative processes.

#### 3.1.1 Curcumin

Curcumin is widely recognized for its interference with inflammation via multiple pathways (Moon, 2024). In preclinical OA studies, Curcumin significantly improved pathological changes in monosodium iodoacetate (MIA)-induced knee OA. Treatment reduced synovial TNF-α, IL-1α, and IL-6 levels, and inhibited IκBα phosphorylation and NF-κB activation, thereby alleviating synovitis, delaying cartilage degeneration, and improving pain behavior (Wang et al., 2019).

Notably, beyond inhibiting pro-inflammatory signals, Curcumin actively remodels immune cell composition. In collagen-induced arthritis (CIA) models, it not only downregulated NF-κB-dependent inflammatory expression but also induced apoptosis in pathological synovial macrophages, reducing the cellular source of inflammatory mediators (Zhang and Zeng, 2019). This dual mechanism of signal inhibition and inflammatory cell clearance provides key insights into Curcumin’s role in remodeling the osteoimmune microenvironment.

#### 3.1.2 Apigenin

Apigenin plays multiple roles in regulating the balance of bone remodeling (Chen J. et al., 2025). Within the synovial microenvironment, it demonstrates a unique ability to block intercellular crosstalk. A Transwell co-culture study revealed its mechanism: Apigenin primarily targets the TRPM7-mechanistic target of rapamycin (mTOR) axis in synovial macrophages rather than acting directly on cartilage. By inhibiting TRPM7-mediated calcium influx and downstream mTOR phosphorylation, Apigenin successfully blocks macrophage polarization toward the pro-inflammatory M1 phenotype, thereby reducing the paracrine release of IL-1α and TNF-α. This “upstream interception” strategy effectively halts the transmission of inflammatory signals to chondrocytes, preserving cartilage matrix integrity (Ji et al., 2023).

In summary, these studies indicate that the natural product not only regulates osteoimmune homeostasis by inhibiting inflammatory mediators, but also actively remodels macrophage phenotypic plasticity and intercellular crosstalk within the synovial niche.

### 3.2 Bridging innate and adaptive immunity: dual-regulation of macrophages and T Cells

OA is primarily driven by innate immunity, while RA is mainly driven by adaptive immunity. However, given the extensive crosstalk between innate and adaptive immunity, natural products targeting both macrophages and T cells offer synergistic therapeutic advantages. Although direct evidence in OA models is emerging, extensive studies in RA provide a compelling mechanistic blueprint for how these compounds restore the Th17/Treg rheostat. Although OA lacks the autoantigen-driven pathology characteristic of RA, accumulating evidence indicates that shared immunometabolic checkpoints—such as AMPK–mTOR signaling, Th17/Treg plasticity, and macrophage–T cell crosstalk—are conserved across chronic inflammatory joint diseases, supporting cautious mechanistic extrapolation. Nevertheless, future research urgently needs to validate efficacy using OA-specific models.

#### 3.2.1 Berberine

Berberine acts as a metabolic-immune switch. In CIA models, Berberine significantly inhibited the activation of CD4^+^ Th cells and CD4+CXCR5+ Tfh cells, reducing the expression of co-stimulatory molecules CD28 and CD154 (CD40L). Concurrently, Berberine reshaped the CD4^+^ T cell subset balance, shifting the Th17/Treg equilibrium toward Foxp3+ Treg cells, thereby delaying disease onset (Cui et al., 2018; Vita et al., 2021). Mechanistically, this immunomodulation is likely mediated by regulating gut microbiota and activating AMPK (Yu et al., 2020), which antagonizes the mTOR-HIF-1α pathway required for Th17 glycolysis (Wang Y. et al., 2024). In the context of OA, this suggests Berberine has the potential to serve as a “metabolic checkpoint inhibitor,” restraining the pathogenic metabolic reprogramming of synovial T cells.

#### 3.2.2 Celastrol

Similarly, Celastrol targets the core transcriptional machinery of T cell differentiation. In adjuvant-induced arthritis (AA) rat models, it regulated the balance between pathogenic Th17 and protective Treg cells in the joint. Celastrol reduced Th17 cells and increased Treg cells by inhibiting pSTAT3 activation, while stabilizing Foxp3 expression in arthritic joints (Venkatesha et al., 2011; Astry et al., 2015). By blocking the IL-6/STAT3 axis, Celastrol not only suppresses the expansion of pathogenic Th17 cells but also prevents the conversion of Tregs into pro-inflammatory “ex-Tregs” within the cytokine-rich synovial microenvironment.

These findings support the view that adaptive immune regulation in OA should not be interpreted as classical immunosuppression similar to that in RA, but rather as a correction of chronic low-grade immune metabolic imbalance.

#### 3.2.3 Sinomenine

The Aryl Hydrocarbon Receptor (AhR) is a key pathway for inducing immune tolerance (Pernomian et al., 2020). Sinomenine offers a unique mechanism involving AhR agonism. Studies in CIA mice show that Sinomenine functions as an AhR agonist, promoting the generation of functional Treg cells and suppressing Th17 responses in gut-associated lymphoid tissues, thereby alleviating joint inflammation via the gut-joint axis (Tong et al., 2016; Tong et al., 2015). This implies that Sinomenine could ameliorate OA synovitis not only locally but by resetting systemic immune tolerance.

### 3.3 Blockade of inflammatory cascades: NF-κB and NLRP3 inflammasome

Chronic activation of inflammatory pathways, including NF-κB, MAPK, and JAK/STAT, leads to persistent cytokine production in OA. Natural products can block these cascades at multiple nodes, reducing the expression of catabolic and inflammatory genes. This multi-target inhibition not only limits cytokine overproduction but also alleviates feedback loops that exacerbate joint degeneration.

#### 3.3.1 Celastrol

Beyond its effects on T cells, Celastrol exhibits potential in blocking upstream inflammatory triggers in innate immunity (Lei et al., 2025). While most studies focus on TLR4, recent research identified that TLR2 is significantly upregulated in OA chondrocytes and acts as a key driver of NF-κB phosphorylation. Celastrol was confirmed to specifically block the TLR2/NF-κB signaling axis, thereby inhibiting the downstream COX-2 and PGE2 cascade. Crucially, its protective effects extend to the subchondral bone, significantly reducing osteophyte formation and pathological bone resorption in MCLT rat models (Yang et al., 2022). This demonstrates that by targeting TLR2-mediated innate immune recognition, Celastrol can quell cartilage inflammation and remodel the compromised subchondral bone microenvironment. Additionally, recent RNA-seq studies identified Pristimerin as a multi-target regulator. Unlike single-target drugs, Pristimerin simultaneously inhibits NF-κB and MAPK signaling. This dual inhibition effectively reverses IL-1α-induced upregulation of catabolic mediators (MMP-13) and inflammatory enzymes (iNOS, COX-2), preserving Collagen II expression and mitigating cartilage degeneration in destabilization of the medial meniscus (DMM) mice (Yin et al., 2024).

#### 3.3.2 Sinomenine

Sinomenine (SIN) delays OA progression by inhibiting inflammatory cytokine expression and chondrocyte apoptosis (Huang et al., 2022).

While inhibiting NF-κB is a primary strategy, targeting the NLRP3 inflammasome offers a novel route to halt the inflammatory cascade. SIN regulates inflammation through a unique epigenetic mechanism. Rather than directly blocking kinase activity, SIN significantly upregulates the expression of miR-223-3p in chondrocytes. This specific microRNA acts as a post-transcriptional repressor, targeting and degrading NLRP3 mRNA. By disrupting NLRP3 inflammasome assembly, SIN not only reduces IL-1α and IL-18 maturation but also effectively prevents inflammation-induced chondrocyte apoptosis in anterior cruciate ligament transection (ACLT) models (Dong et al., 2019). This highlights the potential of natural products as “epigenetic regulators” in restoring the osteoimmune microenvironment.

On the other hand, SIN shows significant efficacy in restoring redox balance. Recent studies using DMM mouse models reveal its key role in activating the Nrf2/HO-1 signaling axis. Under inflammatory stimulation, SIN promotes Nrf2 nuclear translocation, upregulating antioxidant enzymes like HO-1. Interestingly, this study highlighted molecular crosstalk, where SIN-induced Nrf2 activation is accompanied by simultaneous NF-κB suppression (Wu et al., 2019). This suggests SIN functions as a “molecular switch,” transitioning chondrocytes from a pro-inflammatory/oxidative state to an anti-inflammatory/antioxidant state.

In summary, it can be understood that the natural product does not target a single cytokine, but rather simultaneously blocks multiple inflammatory amplification circuits in the osteoimmune microenvironment.

### 3.4 Reversing immune-driven metabolic reprogramming: targeting the HIF axis

In the osteoimmune microenvironment, the metabolic reprogramming of chondrocytes is not a spontaneous cellular event, but is profoundly driven by the persistent inflammatory cytokine network (e.g., IL-1β and TNF-α derived from M1 macrophages). Given the critical role of this immune-driven HIF switch in OA pathology, restoring physiological hypoxic responses is a new therapeutic Frontier. Natural products have demonstrated the capacity to correct the imbalance between HIF-1α and HIF-2α. By realigning this “metabolic switch,” natural products can shift chondrocytes from a catabolic, apoptotic state back to an anabolic, homeostatic state.

#### 3.4.1 Apigenin

In addition to its immunomodulatory effects (Section 3.1.2), Apigenin demonstrates remarkable efficacy in directly reshaping chondrocyte metabolism. It exhibits a unique “dual-target” mechanism. Transcriptionally, it acts as a potent inhibitor of HIF-2α. By blocking the upstream JNK/NF-κB cascade, Apigenin suppresses HIF-2α-driven upregulation of catabolic enzymes (MMP-13, ADAMTS-5), effectively turning off the catabolic switch (Cho et al., 2019). Bioenergetically, Apigenin targets CD38, a key NADase overexpressed in OA. Inhibiting CD38 restores the intracellular NAD+:NADH ratio, crucial for Sirtuin activity and mitochondrial function. This metabolic restoration not only reduced cartilage degeneration but also alleviated pain in DMM mice, highlighting the therapeutic value of targeting the CD38-NAD + axis (Gil et al., 2023).

#### 3.4.2 Icariin

Icariin, a prenylated flavonoid from Epimedium, exerts significant positive effects on bone metabolism and remodeling (Luo et al., 2025).

While Apigenin restores cellular “fuel” (NAD+), Icariin directly acts as an ignition for the metabolic “engine,” sirtuin 1 (SIRT1). As an NAD+-dependent deacetylase, SIRT1 is a key sensor linking cellular energy status to stress resistance. Studies using si-SIRT1 knockdown showed that Icariin’s protective effects are dependent on SIRT1. By upregulating SIRT1, Icariin triggers the downstream Nrf2/HO-1 axis, bridging metabolic sensing and antioxidant defense. Furthermore, this metabolic regulation preserves genomic stability (evidenced by reduced DNA damage in comet assays) (Liu et al., 2024).

In the hypoxic environment of OA, the balance between HIF isoforms often determines cell fate. While Apigenin targets the pathological HIF-2α, Icariin acts as a specific activator for the regenerative isoform HIF-1α. As a phytoestrogen, Icariin was shown to upregulate HIF-1α expression in bone marrow stromal cells (BMSCs) via the estrogen receptor (ER). Stabilization of HIF-1α did not trigger catabolism but instead activated the CXCR4/SDF-1α axis, significantly enhancing the migration and homing of progenitor cells to injury sites (Zhu et al., 2018).

In summary, Apigenin and Icariin represent a complementary therapeutic strategy: one blocks the catabolic switch (HIF-2α) in chondrocytes, while the other turns on the “repair switch” (HIF-1α) in progenitors, jointly reprogramming the hypoxic response toward tissue regeneration. Therefore, immune-driven metabolic remodeling holds promise as a key link between osteoimmune dysregulation and chondrocyte degeneration.

### 3.5 Mitigation of immune-mediated oxidative stress and ferroptosis

Within the OA osteoimmune microenvironment, oxidative stress is primarily fueled by the local inflammatory cascade. Cytokines secreted by polarized macrophages and Th17 cells continuously stimulate chondrocytes to overproduce ROS, thereby accelerating lipid peroxidation and subsequent ferroptosis. Following the restoration of immune-metabolic homeostasis, natural products also exert profound effects on these downstream consequences (Yu et al., 2025; Xu et al., 2024; Gu et al., 2024). Natural products primarily combat this immune-mediated oxidative stress by activating the Nrf2/HO-1 pathway, enhancing endogenous antioxidant defenses.

#### 3.5.1 Sulforaphane

Sulforaphane (SFN) possesses broad activity (Hajimohammadi et al., 2024). While many antioxidants directly scavenge free radicals, SFN adopts a more complex “master regulator” strategy. A mechanistic study demonstrated that SFN activates TFEB, a master regulator of lysosomal function, via a mechanism that is Ca2+-dependent but mTOR-independent. Interestingly, TFEB was found to directly control the expression of NFE2L2/NRF2, placing autophagy upstream of antioxidant defense. By triggering the TFEB-NRF2 axis, SFN simultaneously enhances the expression of detoxification enzymes (via ARE) and accelerates the clearance of damaged mitochondria through autophagy. This “dual defense” mechanism enables chondrocytes to effectively mitigate aging-related acute oxidative bursts and chronic oxidative stress (Li et al., 2021).

Notably, this ability to restore redox homeostasis via the Keap1/Nrf2 pathway is not unique to Sulforaphane but is widely present in various dietary natural products. A study on human chondrocyte samples compared the effects of Allicin, Lycopene, and Sulforaphane. The results showed that all three compounds could effectively activate the Keap1/Nrf2 signaling axis, thereby upregulating the expression of antioxidant enzymes. The study also revealed a key cytoprotective mechanism: in addition to anti-apoptosis, these antioxidants could significantly inhibit oxidative stress-induced chondrocyte hypertrophic differentiation. Since cartilage hypertrophy is a hallmark event in the development of OA towards late-stage ossification, this suggests that dietary supplementation with Nrf2 activators can not only scavenge ROS but also maintain the healthy phenotype of chondrocytes and promote matrix synthesis (Yang et al., 2020).

#### 3.5.2 Epigallocatechin-3-gallate

Epigallocatechin-3-gallate (EGCG) is a highly bioactive polyphenol renowned for its potent antioxidant, anti-inflammatory, and anti-apoptotic properties (Darweish et al., 2014; Alatawi et al., 2025). As a representative tea polyphenol, EGCG exhibits multi-level regulatory capabilities in maintaining chondrocyte redox homeostasis. Regarding classic antioxidant pathways, EGCG has been confirmed to dissociate the inhibition of Keap1 on Nrf2. A reverse validation study using an Nrf2-specific inhibitor demonstrated that EGCG must exert its effects by activating the Nrf2/HO-1/NQO1 axis. This activation not only cleared ROS but also significantly inhibited cellular senescence. In addressing novel forms of cell death, improved nanodrug formulations of EGCG (ES NDs) further demonstrated potential in combating ferroptosis (Zhu et al., 2022).

Therefore, redox regulation represents a downstream convergence point, through which immune activation ultimately leads to ferroptosis susceptibility.

### 3.6 Restoration of immune-suppressed autophagy to prevent apoptosis

Autophagy is a protective mechanism within chondrocytes that removes damaged organelles and proteins to prevent apoptosis. However, in the OA joint, the autophagic flux in chondrocytes is not solely impaired by intrinsic aging, but is profoundly suppressed by the pro-inflammatory chronic cytokine imbalance orchestrated by M1 macrophages and aberrant T cells. This chronic immune signaling overactivates the mTOR pathway and inhibits AMPK. Various natural products can restore autophagy function, promoting cell survival. By strengthening this cellular defense function, natural products can not only prevent apoptosis but also indirectly regulate inflammatory and oxidative stress responses, thereby achieving network regulation within the microenvironment.

#### 3.6.1 Berberine

Berberine has been confirmed to have positive therapeutic effects and broad application prospects in the pathological changes of human-related metabolic and chronic diseases (Wang K. et al., 2024; Wong et al., 2020). Autophagy is strictly regulated by the mutual influence between AMPK and mTOR, which integrate cellular energy status and nutrient supply. Berberine has been identified as a specific activator of this key metabolic checkpoint. A landmark study using AMPKα1 knockout mice provided conclusive evidence for its mechanism. The research found that Berberine induces AMPK phosphorylation via its upstream kinase LKB1. This activation is crucial for restoring the expression of downstream metabolic regulators SIRT1 and SIRT3, which are key to mitochondrial quality control. Importantly, the protective effects of Berberine against cartilage degeneration, synovitis, and pain observed in wild-type mice were completely abolished in AMPKα1 knockout mice (Li J. et al., 2022). This confirms that Berberine rescues chondrocytes from metabolic dysfunction and catabolic stress strictly through the LKB1-AMPK axis. Beyond metabolic regulation, Berberine’s protective network involves critical crosstalk with stress signaling pathways. A study using an SNP-induced apoptosis model showed that Berberine-mediated AMPK activation serves as a negative regulator of the p38 MAPK pathway. By chemically blocking AMPK, the research confirmed that Berberine requires AMPK to inhibit p38 phosphorylation. This signaling regulation translates into tangible morphological protection: Berberine significantly inhibits maladaptive cytoskeletal remodeling and restores the Bcl-2/Bax ratio, effectively preventing Caspase-3 dependent apoptosis (Zhou et al., 2015). Overall, these findings indicate that Berberine protects chondrocytes by coordinating a shift from “catabolic stress” (p38 MAPK) to “energy survival” (AMPK/SIRT1).

#### 3.6.2 Resveratrol

Resveratrol is a natural polyphenol compound with anti-inflammatory and antioxidant properties, which has received attention in recent years as a candidate drug for treating OA (Hou et al., 2025). Resveratrol can prevent the occurrence of OA and show certain therapeutic effects by inhibiting inflammatory responses, protecting chondrocytes, maintaining cartilage homeostasis, and promoting autophagy. This process may be related to the regulation of signaling pathways such as Nuclear Factor-κB (NF-κB), Toll-like Receptor 4 (TLR4), and SIRT1 (Zou et al., 2024). Beyond mitochondrial dysfunction, Endoplasmic Reticulum (ER) stress has become a key driver of chondrocyte apoptosis, especially in aging- and obesity-related OA. Persistent ER stress leads to the accumulation of unfolded proteins, triggering the upregulation of the pro-apoptotic transcription factor CHOP and leading to blocked autophagy flux. A recent study utilizing a novel ER stress-driven OA mouse model (I-ERS mice) demonstrated that Resveratrol alleviates ER stress-induced autophagy blockade by inhibiting the expression of CHOP and the senescence marker p16INK4a. This restoration of organelle homeostasis significantly reduced the number of TUNEL-positive chondrocytes and preserved proteoglycan content, highlighting the potential of targeting the ER stress-autophagy axis to prevent OA (Hecht et al., 2021).

The efficacy of Resveratrol in resolving ER stress was further embodied in a severe genetic model of chondrodysplasia (PSACH). In MT-COMP mice, misfolded COMP proteins accumulate in the ER, triggering a vicious cycle of CHOP-mediated stress and mTORC1-driven autophagy blockade. Resveratrol treatment suppresses inflammation and reactivates autophagy, thereby promoting the clearance of mutant proteins from the ER and breaking this vicious cycle. This indicates that Resveratrol is not merely a symptom reliever but a “proteostasis regulator” capable of correcting fundamental organelle dysfunction in stressed chondrocytes (Hecht et al., 2023).

#### 3.6.3 Matrine

Notably, mitochondrial dysfunction often constitutes a “Vicious Cycle Network (VCN)” involving bioenergetic failure and quality control defects. A comprehensive review emphasized that alkaloids such as matrine and its derivatives can simultaneously engage multiple nodes of this network to restore redox balance and energy metabolism (He et al., 2025).

In addition to activating positive regulators like AMPK and SIRT1, relieving the negative inhibition of autophagy is an equally effective strategy. Oxymatrine (OMT), a quinolizidine alkaloid, functions by directly targeting the AKT/mTOR signaling axis. In IL-1β-treated chondrocytes, OMT treatment significantly inhibits the phosphorylation of AKT and mTOR, thereby relieving the inhibition of autophagy. The restoration of autophagy flux is accompanied by reduced intracellular ROS levels and protection against matrix degradation. Crucially, the chondroprotective effect of OMT is abolished by the autophagy inhibitor 3-MA, confirming that mTOR-mediated autophagy is an indispensable mechanism of action (Lu et al., 2024).

Therefore, restoring autophagy flux enables chondrocytes to regain resilience against persistent osteoimmune stress.

### 3.7 Restoring the subchondral bone microenvironment via immune-bone crosstalk

Pathological changes in OA are not limited to articular cartilage; abnormal remodeling of the subchondral bone plays an equally crucial role in disease progression (Liang et al., 2024; Hu Y. et al., 2021). Importantly, this pathological bone remodeling is deeply intertwined with synovial inflammation through specific ‘immune-bone crosstalk.’ Pro-inflammatory cytokines (e.g., IL-6, TNF-α) and aberrant RANKL expression derived from the osteoimmune microenvironment excessively activate osteoclasts. These abnormally active osteoclasts accelerate cartilage degeneration from the bottom up via altered biomechanical loads and further cytokine release. Therefore, targeting the subchondral bone microenvironment to restore the balance between osteoblasts and osteoclasts is a key strategy for maintaining overall joint homeostasis (Zhao et al., 2022; Zuo et al., 2024).

Icariin demonstrates remarkable “bone-cartilage” dual protection capabilities in this field. Beyond improving cartilage metabolism as mentioned above, Icariin is a potent regulator of the subchondral bone microenvironment. In an LPS-induced inflammatory osteolysis model, Icariin was confirmed to significantly inhibit osteoclast differentiation and bone resorption activity. Its core mechanism lies in correcting the pathological RANKL/OPG ratio: Icariin downregulates the expression of the osteoclast differentiation factor RANKL while upregulating the levels of the osteoprotegerin OPG.

Mechanistic studies further revealed that this inhibitory effect is achieved by blocking the p38 and JNK MAPK signaling pathways within osteoclasts, as well as inhibiting the expression of the hypoxia-inducible factor HIF-1α (notably, this is opposite to its role in activating HIF-1α in chondrocytes to promote repair, reflecting the regulatory specificity of natural products in different cell types) (Hsieh et al., 2011). By preventing inflammatory bone loss in the subchondral bone, Icariin provides stable structural support for articular cartilage, thereby delaying the overall progression of OA.

### 3.8 Integrated regulation of the osteoimmune microenvironment

Collectively, the mechanisms described above highlight the pleiotropic nature of natural products in regulating the OA microenvironment. Unlike traditional single-target biologics, natural products act as network regulators (Yin et al., 2024; He et al., 2025). They regulate macrophage polarization at the source (Ji et al., 2023; Lee et al., 2025), block inflammatory cascades in the middle (Yang et al., 2022; Dong et al., 2019), scavenge ROS and inhibit ferroptosis at the environmental level (Yang et al., 2020; Yu et al., 2024), and restore autophagy and metabolic checkpoints downstream (Cho et al., 2019; Li J. et al., 2022; Lu et al., 2024).

**FIGURE 2:**
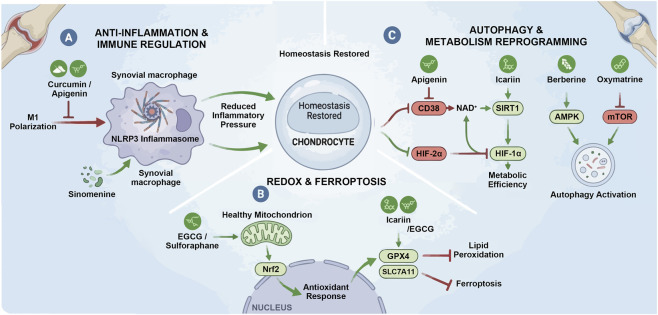
Targeted modulation of the osteoimmune microenvironment by natural products. **(A)** Anti-inflammation and immune regulation, **(B)** redox and ferroptosis, and **(C)** autophagy and metabolism reprogramming. Proposed pathway hierarchy and therapeutic interception: Upstream immune triggers → inflammatory/metabolic stress and redox imbalance → RCD pathways → natural product intervention points.

This holistic intervention strategy, often validated through network pharmacology analysis (Zhao et al., 2023), can effectively reverse the pathological reprogramming of the osteoimmune microenvironment. By simultaneously addressing upstream immune triggers and downstream executors of cell death programs, natural products provide a comprehensive strategy for restoring joint homeostasis. Table 1 comprehensively summarizes natural products and their specific molecular targets within the osteoimmune microenvironment. The orchestrated molecular networks modulated by these natural products are visually summarized in Figure 2.

**TABLE 1:** Natural products targeting key pathways of the osteoimmune microenvironment in osteoarthritis.

Natural product	Source	Target microenvironment component	Molecular targets/Pathways	Mechanism of cytoprotection/Regulation	Model	Phenotypic improvements	Ref.
Curcumin	Curcuma longa	Synovial Macrophage	NF-κB, COX-2	Promotes Macrophage Apoptosis	*In vivo*: CIA Rats *In vitro*: RAW264.7 cells	Alleviated synovitis; Delayed cartilage degeneration; Improved pain behavior	Wang et al. (2019)
Curcumin	Curcuma longa	Synovial Inflammatory Microenvironment	TLR4/MyD88/NF-κB	Inhibits Inflammatory Cytokine Release	*In vivo*: MIA-induced OA rats		Zhang and Zeng (2019)
Apigenin	*Apium graveolens* (Generic)	Macrophage-Chondrocyte Crosstalk	TRPM7/mTOR; MAPK (p38/JNK/ERK)	Restores Osteoimmune Homeostasis: Inhibits M1 polarization to cut off inflammatory signals; Inhibits Chondrocyte Apoptosis via paracrine regulation	*In vivo*: Modified Hulth mice *In vitro*: Transwell Co-culture	Reduced synovial hyperplasia; Preserved cartilage matrix integrity	Ji et al. (2023)
Berberine	Rhizoma Coptidis	Synovial T Cells (Th17/Treg balance)	AMPK; mTOR-HIF-1α; CD28/CD154	Metabolic-Immune Switch: Activates AMPK to antagonize mTOR-HIF-1α mediated Th17 glycolysis; Modulates gut microbiota to promote Foxp3+ Treg differentiation	*In vivo*: CIA Mice; DSS-induced UC mice	Delayed arthritis onset; Reduced joint swelling and systemic inflammation	Cui et al. (2018), Vita et al. (2021)
Celastrol	Tripterygium wilfordii	Synovial T Cells (Th17/Treg balance)	IL-6/STAT3; pSTAT3	Transcriptional Regulation: Blocks STAT3 phosphorylation to inhibit Th17 lineage commitment; Stabilizes Foxp3 expression to prevent Treg plasticity	*In vivo*: Adjuvant-induced Arthritis (AA) Rats	Reduced osteophyte formation; Decreased pathological subchondral bone resorption	Venkatesha et al. (2011), Astry et al. (2015)
Sinomenine	Sinomenium acutum	Systemic & Synovial T Cells	Aryl hydrocarbon receptor	Gut-Joint Tolerance Reset: Acts as an AhR agonist to induce intestinal Treg generation; Promotes Treg trafficking from gut to joint to suppress Th17 responses	*In vivo*: CIA Mice/Rats	Attenuated cartilage destruction; Improved joint mobility	Tong et al. (2016), Tong et al. (2015)
Celastrol	Tripterygium wilfordii	Chondrocyte & Subchondral Bone	TLR2/NF-κB	Suppresses inflammatory mediators (COX-2, PGE2); Inhibits Pattern Recognition Receptor (TLR2); Reduces Bone Resorption & Osteophytes	*In vivo*: MCLT+pMMT Rats *In vitro*: Human/Rat Chondrocytes	Delayed cartilage damage, suppressed the production of inflammatory factors	Yang et al. (2022)
Pristimerin	Tripterygium wilfordii	Chondrocyte (Inflammatory state)	NF-κB, MAPK; iNOS/COX-2	Inhibits Inflammatory Cascades; Suppresses Catabolism (Reverses MMP-13 upregulation); Restores Col II	*In vivo*: DMM mice *In vitro*: IL-1β induced chondrocytes + RNA-seq	Reduced cartilage degradation, slowed OA progression	Yin et al. (2024)
Sinomenine	Sinomenium acutum	Chondrocyte	miR-223-3p; NLRP3	Epigenetic Regulation: Targets miR-223-3p to block NLRP3 assembly; Inhibits Pyroptosis/Apoptosis	*In vivo*: ACLT mice (Post-traumatic OA); *In vitro*: IL-1βchondrocytes	Attenuated cartilage destruction; Improved joint mobility	Dong et al. (2019)
Sinomenine	Sinomenium acutum	Chondrocyte (Oxidative Stress state)	Nrf2/HO-1; NF-κB	Restores Redox Homeostasis; Crosstalk Regulation (Balances oxidation and inflammation); Inhibits Catabolism (MMP/ADAMTS)	*In vivo*: DMM mice *In vitro*: IL-1 chondrocytes		Wu et al. (2019)
Apigenin	Cirsium japonicum/Generic	Chondrocyte	HIF-2α(JNK/NF-κB)	Reverses Metabolic Reprogramming 1. Blocks Catabolic Switch: Inhibits MMP3/13 and ADAMTS4	*In vivo*: DMM mice (WT and CD38 KO)	Prevented cartilage erosion; Alleviated mechanical allodynia (pain)	Cho et al. (2019)
Apigenin	Cirsium japonicum/Generic	Chondrocyte	CD38 (NADase)	2. Restores Energy Homeostasis: Normalizes NAD+:NADH ratio 3. Alleviates Pain: Reduces synovial CGRP expression	*In vitro*: Human/Mouse cells		Gil et al. (2023)
Icariin	Epimedium	Chondrocyte (Metabolic/Genomic Stress)	SIRT1 (NAD+) Nrf2	Restores Metabolic & Genomic Stability: Activates SIRT1 to drive downstream antioxidant defense and DNA repair; Validated by si-SIRT1	*In vivo*: ACLT mice; *In vitro*: IL-1β induced	Prevented inflammatory bone loss; Maintained bone-cartilage unit integrity	Liu et al. (2024)
Icariin	Epimedium	Progenitor Cells (BMSCs in Joint)	HIF-1α; CXCR4	Promotes Hypoxic Adaptation & Repair: Stabilizes HIF-1αto drive CXCR4-mediated migration/homing of repair cells	*In vitro*: BMSCs (with si-HIF-1 validation)		Zhu et al. (2018)
Berberine	Coptis chinensis (Generic)	Chondrocyte & Synovial Microenvironment	AMPK (LKB1); SIRT1/3	Restores Metabolic Homeostasis; Inhibits Pain & Synovitis; Validated by AMPKα1 KO	*In vivo*: DMM mice (WT and AMPKα1 KO) *In vitro*: Human Chondrocytes	Rescued cartilage structural damage; Relieved joint pain	Li et al. (2022c)
Berberine	Coptis chinensis	Chondrocyte (SNP-induced Apoptosis)	AMPK p38 MAPK; iNOS	Inhibits Apoptosis (Regulates Bcl-2/Bax, Caspase-3); Preserves Cytoskeleton Integrity	*In vivo*: Surgical OA Rat; *In vitro*: SNP-stimulated cells		Zhou et al. (2015)
Resveratrol	Vitis vinifera (Grapes)	Chondrocyte (Under ER Stress)	ER Stress/CHOP; pS6 (mTOR); MMP-13	Relieves ER Stress-induced Autophagy Block; Inhibits CHOP-mediated Apoptosis & Senescence (p16)	*In vivo*: I-ERS mice (ER stress-driven model)	Preserved proteoglycan content; Significantly reduced TUNEL-positive (dead) chondrocytes	Hecht et al. (2021)
Resveratrol	Vitis vinifera	Chondrocyte (Severe ER Stress)	CHOP; mTORC1; Autophagy	Promotes Mutant Protein Clearance; Defines “Therapeutic Window” for intervention; Relieves ER stress-induced inflammation	*In vivo*: MT-COMP mice (PSACH model)		Hecht et al. (2023)
Oxymatrine	Sophora flavescens	Chondrocyte (Autophagy-deficient)	AKT/mTOR; ROS	Relieves mTOR-mediated Autophagy inhibition; Reduces Apoptosis & Matrix Degradation (Validated by 3-MA)	*In vitro*: Rat Chondrocytes (IL-1 induced)	Reduced matrix degradation; Protected chondrocyte viability	Lu et al. (2024)
Icariin	Epimedium	Osteoclast (in Subchondral Bone)	RANKL/OPG ratio; HIF-1α; p38/JNK	Inhibits Osteoclastogenesis: Suppresses bone resorption and osteolysis; Prevents inflammatory bone loss	*In vitro*: LPS-induced Osteoclasts	Prevent inflammatory bone loss	Hsieh et al. (2011)
Curcumin (dCOL2-CM-Cur-PNPs)	Curcuma longa (Engineered)	Damaged Cartilage Matrix & Synovial Macrophage	M1/M2 Polarization; Chondrogenic genes	Targeted Delivery: Specifically binds to dCOL2; Immunomodulation: Inhibits M1 and Promotes M2; Sustained release	*In vivo*: DMM Rats *In vitro*: iMSC-CMs	Enhanced targeted accumulation in inflamed joints; Accelerated cartilage repair	Lee et al. (2025)
EGCG-SeMet Nanodrugs (ES NDs)	Camellia sinensis (Green tea) + Selenium	Chondrocyte (Ferroptosis state)	GPX4; Lipid Peroxidation; Fe2+ accumulation	Inhibits Ferroptosis (Iron-dependent cell death); Nanodrug enhances bioavailability	*In vivo*: DMM mice (Intra-articular) *In vitro*: Oxidative stress model	Reduced iron accumulation in joints; Improved weight-bearing distribution (joint function)	Yu et al. (2024)

Table 2 also provides a targeted mechanistic map. This atlas elucidates how different osteoimmune processes directly trigger the regulated cell death of specific subtypes of chondrocytes and identifies the corresponding natural products that can block these exact pathways.

**TABLE 2:** Osteoimmune triggers of chondrocyte regulated cell death and representative natural products.

Osteoimmune trigger/Driver	Mediating signaling pathway	Chondrocyte RCD subtype	Targeting natural product
M1 macrophage polarization	NF-κB/TNF-α	Apoptosis	Curcumin
Th17/Treg imbalance	IL-17/STAT3	Apoptosis/Pyroptosis	Berberine
NLRP3 inflammasome activation	Caspase-1/GSDMD	Pyroptosis	Sinomenine
Immune-mediated ROS accumulation	Lipid peroxidation	Ferroptosis	EGCG
Immune-driven HIF-2α shift	Mitochondrial dysfunction	Apoptosis	Apigenin

## 4 Current challenges and future strategies

The medicinal use of natural products dates back to around 2600 BC, as evidenced by Sumerian cuneiform tablets from Mesopotamia listing over a thousand plants and resins used clinically (Ne and wman, 2022). Today, the global use of natural products continues to rise (Butler et al., 2026). Although the mechanisms detailed in Chapter three depict a promising landscape, a significant gap remains between preclinical success and clinical translation, with application hurdles such as pharmacokinetics and targeted delivery still unresolved (Tang et al., 2025). Consequently, no natural product has yet been approved as a first-line drug for OA alleviation (Kolasinski et al., 2020). This section dissects these barriers and proposes engineering solutions to cross this “Valley of Death” that has spanned millennia.

### 4.1 The pharmacokinetic paradox and therapeutic window

While natural products demonstrate robust efficacy in most animal and cell experiments, a major obstacle in practical application is the poor pharmacokinetic profile of most natural compounds. As emphasized by Xu et al., polyphenols such as curcumin and resveratrol undergo rapid metabolism and systemic elimination, resulting in negligible intra-articular concentrations following oral administration (Xu et al., 2025). Even with intra-articular injection, small-molecule alkaloids are cleared from the synovial space via lymphatic drainage within hours, failing to maintain the therapeutic duration required for cartilage repair. Furthermore, the timing of intervention is often overlooked. Evidence from genetic OA models (MT-COMP mice) concurrently indicates that natural product treatment must adhere to a strict therapeutic window; resveratrol intervention is effective only when initiated early postnatally but fails once structural degeneration has occurred (Hecht et al., 2023). This implies that natural products may function best as prophylactic agents or in early-stage OA, providing new insights into their clinical positioning. Therefore, superior biomarkers and novel detection methods are required to enable early diagnosis in clinical trials (Chen Y. et al., 2025; Shen et al., 2023).

### 4.2 Engineering breakthroughs: nanodelivery systems

**FIGURE 3:**
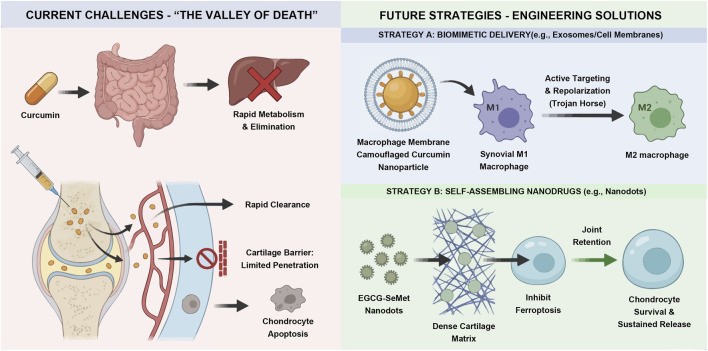
Current challenges and future nanomedicine strategies for natural product delivery.

Nanodelivery systems involve loading drugs into nanoscale carriers to achieve precise regulation of the disease microenvironment by improving drug stability, bioavailability, and targeting (Yao et al., 2025; Wu et al., 2024). As aforementioned, despite their potent biological activity, most natural products suffer from poor solubility and limited bioavailability, severely restricting their clinical translation in OA. Thus, there is an urgent need for alternative administration routes to circumvent the barriers associated with intra-articular injection (Mao et al., 2021). Advanced drug delivery systems (DDS) based on nanocarriers, characterized by their small size, tunable targeting, and site-specificity, hold promise for resolving these drawbacks (Huang et al., 2024) (Figure 3).

Biomimetic Delivery: A recent breakthrough involves encapsulating curcumin in engineered vesicles derived from biomimetic stem cell membranes. These biomimetic nanoparticles (Cur-NPs) not only evade immune clearance but also exhibit “homing” capabilities, actively targeting synovial inflammation and repolarizing M1 macrophages to the M2 phenotype (Lee et al., 2025). This demonstrates that enhancing the cellular uptake of natural ingredients through advanced delivery systems is a viable pathway for achieving precise osteoimmune regulation (Talebian et al., 2023).

Self-Assembling Nanodrugs: Chemical engineering also offers solutions. For instance, EGCG, the major polyphenol in green tea, was successfully assembled with selenium into stable nanospheres (ES NDs). This structural modification protects EGCG from oxidation and significantly improves its cellular uptake, enabling it to effectively target the ferroptosis machinery, glutathione peroxidase 4 (GPX4) in chondrocytes (Yu et al., 2024).

Intra-articular Depots: A groundbreaking study constructed a Rhein-based hydrogel system to encapsulate extracellular vesicles derived from Spirulina (SP-EVs). This system possesses unique pH-responsiveness, capable of sensing the acidic microenvironment of the OA joint cavity and specifically releasing its payload. Uniquely, the hydrogel carrier itself possesses anti-inflammatory activity, generating a synergistic effect with SP-EVs. This “microalgae-herbal” hybrid system not only achieves sustained delivery but also effectively rescues mitochondrial dysfunction and replenishes ATP levels in chondrocytes by modulating the JAK/STAT pathway, providing a precise engineering solution for reversing metabolic reprogramming (Liang et al., 2025).

This article only briefly describes a few solutions targeting the bone microenvironment through novel engineering approaches. To date, similar solutions are still under exploration, representing another shift from passive drug delivery to active osteoimmune modulation in treatment.

### 4.3 Future directions: precision and combination

The pleiotropy of natural products makes them ideal candidates for Combination Therapy. Rather than relying on a single compound, leveraging the mechanistic complementarity of different natural products to generate synergistic effects is a superior strategy. For example, combining an anti-catabolic agent with an anabolic promoter could accelerate tissue repair while blocking cartilage degradation, offering overall benefits superior to monotherapy. However, for natural products to achieve true clinical translation, the core obstacle in past DMOAD development—disease heterogeneity—must be overcome (Moulin et al., 2025).

Recent studies have profoundly highlighted that OA is not a single homogeneous disease, but a syndrome of distinct pathological mechanisms. Different OA subtypes are driven by distinct signaling networks: inflammatory OA may be dominated by the NF-κB/NLRP3 pathway, while metabolic OA may be driven by HIF/mTOR imbalance. As noted by Mobasheri et al., the failure of many biologics to achieve expected results in past clinical trials underscores the importance of clinical phenotypes and molecular endotypes—a concept highly likely to pave the way for successful OA therapy (Mobasheri and Loeser, 2024; Cho et al., 2021).

Therefore, future research on natural products should be dedicated to Precision Medicine. By developing specific biomarkers and utilizing spatial analysis techniques (Fan et al., 2024), patients can be stratified into “inflammatory responders” or “metabolic responders,” and subsequently matched with natural products possessing specific mechanisms. This precision stratification based on pathological mechanisms is a key step in crossing the “Valley of Death” in DMOAD development.

Emerging spatial multi-omics platforms, such as high-plex protein–transcriptome co-mapping and spatial tri-omics sequencing (Zhang D. et al., 2025; Liu et al., 2023), now enable simultaneous profiling of immune, metabolic, and epigenetic layers within intact joint tissues. When integrated with spatially resolved functional screening tools like Perturb-DBiT (Fan et al., 2025), these approaches hold promise for dissecting endotype-specific osteoimmune niches and accelerating mechanism-matched natural product discovery in OA.

Beyond classical single-pathway models, emerging evidence indicates that chondrocyte death in OA frequently manifests as PANoptosis—a coordinated cell death program that synchronously engages apoptotic, pyroptotic, and necroptotic effectors via innate immune sensor-driven complexes (PANoptosomes) (Zhu et al., 2026). This integrated framework explains the limited clinical efficacy of single-target inhibitors and underscores the strategic advantage of pleiotropic natural products capable of simultaneously modulating upstream immune sensors and downstream execution hubs. Future mechanistic studies should evaluate whether specific natural products can disrupt PANoptosome assembly or intercept osteoimmune-driven PANoptotic signaling in OA.

### 4.4 Differences in osteoimmunology between OA and RA

As mentioned earlier, OA is primarily driven by innate immunity, while RA is dominated by adaptive immunity. In RA, the main characteristic of osteoimmune dysregulation is the activation of self-antigen-driven adaptive immune cells (including Th1/Th17 cells and B cells), leading to sustained systemic cytokine amplification and osteoclast-mediated bone erosion (Bian et al., 2023). Although the mechanisms of osteoimmunology in RA are well-established, their roles in OA are more subtle. OA-related osteoimmunology is mainly triggered by mechanical injury and cartilage matrix degradation, which triggers innate immune activation through DAMP-mediated signaling pathways, leading to chronic low-grade inflammation and progressive cartilage degeneration (Miller et al., 2020). Unlike RA, which is driven by adaptive immune responses, OA is characterized by macrophage polarization, synovial inflammation, and local cytokine production. Given the limited number of studies on the OA osteoimmune system, this article provides some natural product-mediated regulatory mechanisms of RA osteoimmunology for reference.

However, it is important to note that while there is a broad interplay between innate and adaptive immunity, the strategies for osteoimmune-targeted therapy targeting these two systems differ fundamentally. In RA, the core objective is systemic immunosuppression. This is achieved through the use of traditional synthetic DMARDs (such as methotrexate) or biologics targeting single pro-inflammatory pathways (such as TNF-α, IL-6, and the JAK pathway), aiming to completely disrupt the intense autoimmune cascade and prevent systemic joint destruction. However, this “strong suppression” strategy is often ineffective in OA. Due to the localized and low-grade inflammation characteristic of OA, long-term systemic use of highly potent immunosuppressants can lead to severe side effects and fails to precisely target the core pathological changes in cartilage. Therefore, osteoimmune-targeted therapy for OA tends to focus on “microenvironment remodeling” rather than simple “suppression.” Thus, osteoimmune-targeted therapy for OA may require microenvironment reprogramming rather than broad-spectrum immunosuppression (Varela-Eirin et al., 2018).

In contrast, osteoimmune dysregulation in OA involves an interplay of oxidative stress, metabolic reprogramming, and multiple regulatory cell deaths (RCDs). Therefore, OA therapy emphasizes utilizing the “multi-target network regulation” properties of natural products to restore chondrocyte metabolic homeostasis and autophagy function while suppressing low-grade inflammation. Given the differences between the two, caution is still needed when extrapolating treatment strategies from RA to OA.

## 5 Conclusion

The management of OA is undergoing a transition from mere symptom relief to disease-modifying interventions targeting underlying pathologies. As synthesized in this review, the progression of OA is driven by the complex collapse of the osteoimmune microenvironment, characterized by macrophage polarization, metabolic reprogramming, and organelle dysfunction.

Natural products distinguish themselves from traditional single-target biologics through their pleiotropy. Acting as network regulators, compounds like icariin, apigenin, and berberine can simultaneously address multiple aspects of OA pathology: they extinguish inflammation in the synovium, reconstruct metabolism in chondrocytes, and restore the structure of the subchondral bone. This holistic mechanism offers unique advantages in restoring the homeostasis of the entire joint organ.

However, the translation of these promising agents into clinical practice is currently hindered by various challenges, including limited bioavailability, insufficient pharmacokinetic characterization, and the lack of rigorous long-term safety and large-scale clinical validation. Therefore, although many natural products have demonstrated encouraging osteoimmune-regulatory and cytoprotective effects in preclinical studies, they should currently be regarded as promising therapeutic candidates rather than established disease-modifying osteoarthritis drugs (DMOADs).

The future of natural product-based OA therapy lies, on one hand, in the integration of advanced drug delivery systems to ensure precise targeting within the joint cavity. Furthermore, stratifying patients based on their inflammatory or metabolic phenotypes is crucial for fully unleashing the therapeutic potential of natural products. This review proposes an osteoimmune microenvironment–centered pharmacological paradigm for DMOAD development. With continued mechanistic investigation and rigorous translational validation, natural product-based interventions may represent a valuable avenue for preventing chondrocyte regulated cell death and preserving joint function in osteoarthritis.
